# Photo images, 3D models and CT scanned data of loaches (Botiidae, Cobitidae and Nemacheilidae) of Japan

**DOI:** 10.3897/BDJ.6.e26265

**Published:** 2018-07-06

**Authors:** Yuichi Kano, Jun Nakajima, Takeshi Yamasaki, Jyun-ichi Kitamura, Ryoichi Tabata

**Affiliations:** 1 Kyushu University, Fukuoka, Japan; 2 Fukuoka Institute of Health and Environmental Sciences, Dazaifu, Japan; 3 Yamashina Institute for Ornithology, Konoyama, Japan; 4 Mie Prefectural Museum, Tsu, Japan; 5 Lake Biwa Museum, Kusatsu, Japan

**Keywords:** 3D model, Anatomy, Bone, CT scan, Digital archiving, Fish specimen, Freshwater fishes of Japan, GBIF, Holotype, Loach, Morphology, Open science, Paratype, Skeleton, Undescribed species

## Abstract

**Background:**

Loach is one of the major cypriniform ﬁshes in freshwater habitats of Japan; 35 taxa/clades have, until now, been recognised. Parallel to genetic studies, morphological examinations are needed for further development of loach study, eventually ichthyology and fish biology. Digital archiving, concerning taxonomy, ecology, ethology etc., is one of the progressive challenges for the open science of biology. This paper aimed to online publish photo images, 3D models and CT scanned data of all the known clades of loaches inhabiting Japan (103 individuals in total with several type specimens), contributing to ichthyology and public interest of biodiversity/biology.

**New information:**

Photo images, 3D models and CT scanned data of all the known 35 taxa/clades of loaches inhabiting in Japan were online published at http://ffish.asia/loachesOfJapan and http://ffish.asia/loachesOfJapan3D.

## Introduction

Loach is one of the major cypriniform fishes in freshwater habitats of Japan, being recognised with 23 described species/subspecies and 12 undescribed/undecided clades (Nakajima and Uchiyama 2017) (http://ffish.asia/loachesOfJapan). As well as molecular genetic research (Kitagawa et al. 2001a, Kitagawa et al. 2001b, Kitagawa et al. 2003b, Kitagawa et al. 2003a, Kitagawa et al. 2005, Suzawa 2006, Morishima et al. 2008, Saitoh et al. 2010, Kano et al. 2011, Shimizu et al. 2011, Kano et al. 2012, Nakajima 2012, Nakajima 2016, Nakajima and Suzawa 2016, Nakajima and Uchiyama 2017), morphological examinations are needed for further development of loach study. Digital archiving of fish specimens is one of the progressive challenges in ichthyology for open science (Kanagawa Prefectural Museum of Natural History and National Museum of Nature and Science 1998, Berquist et al. 2012, Kano et al. 2013, Kano et al. 2016). This paper aimed to online publish photo images, 3D models and CT scanned data for all the known taxa/clades of loaches inhabiting Japan (http://ffish.asia/loachesOfJapan3D) for the advances of loach study and ichthyology and furthermore as a challenge of open science for public interests of biology.

## General description

### Purpose

This research aims to

1) list all the known taxa/clades of loaches inhabiting Japan with photo images (http://ffish.asia/loachesOfJapan) and

2) digitalise the specimens of each taxa/clades by 3D models and CT scanning (http://ffish.asia/loachesOfJapan3D).

### Additional information

The dataset was also registered on GBIF (http://ipt.pensoft.net/resource?r=jp_loaches_3dct_models)

## Sampling methods

### Study extent

Photo images and specimens of loaches of Japan were taken in the field and borrowed from museums (see "Collection data").

### Sampling description

The specimens were generally captured by a hand-net in the field. All the specimens were fixed in 10% formalin and preserved in 70% ethanol.

### Step description

**Figure 1.**
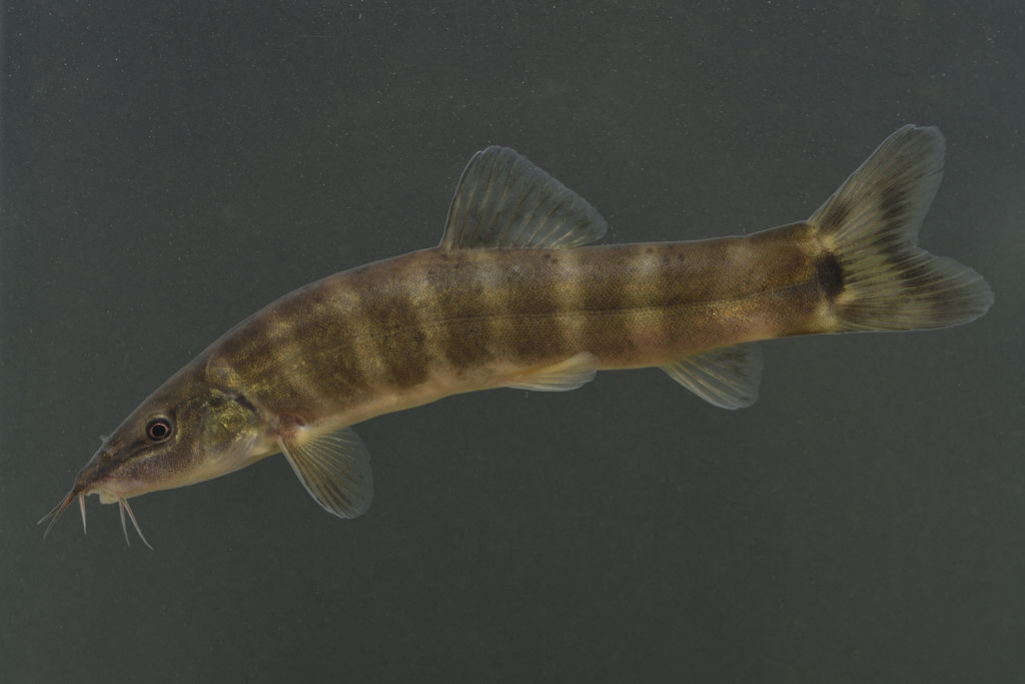
A photo image of an individual of Parabotia curtus in a makeshift aquarium at a wild habitat.

**Figure 2.**
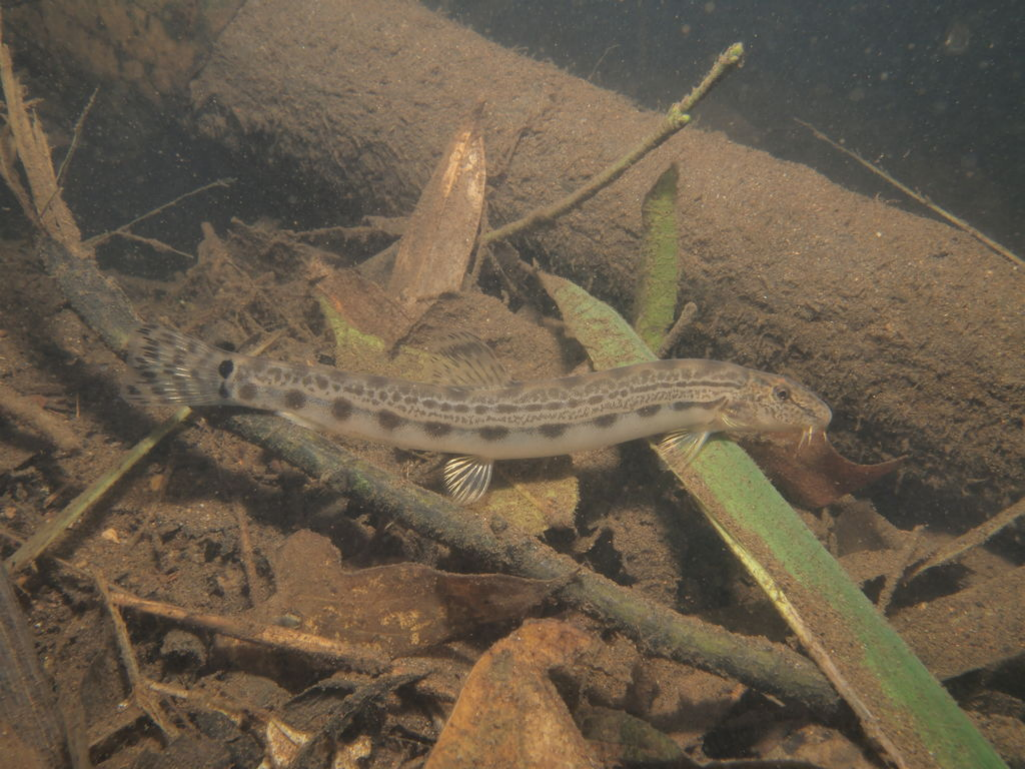
A photo image of Cobitis sakahoko in the wild, under cloudy water by volcanic ash of Mt. Kirishima.

**Figure 3.**
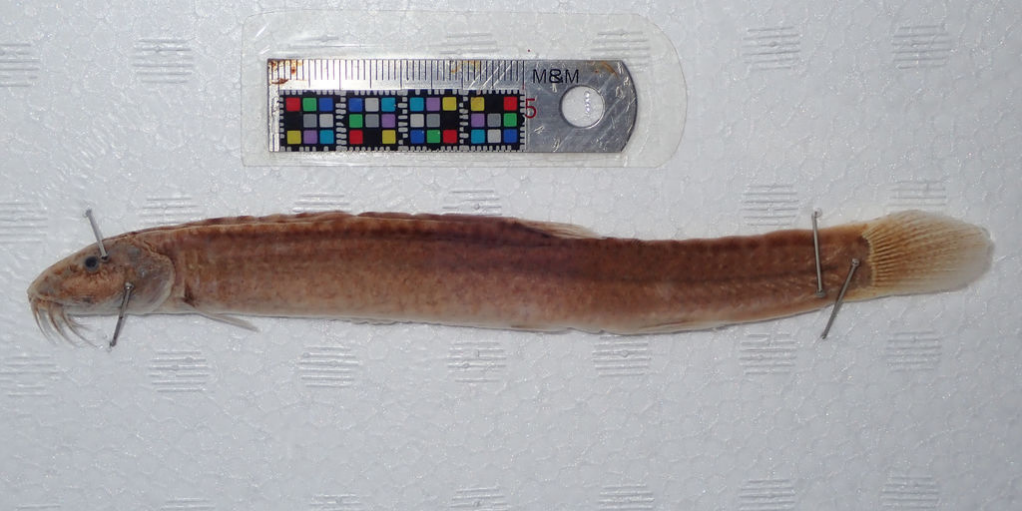
An old specimen of Misgurnus anguillicaudatus (Jindai-dojyô) (MPMQ-JJ1), an uncertain local clade that is already extinct.

**Figure 4.**
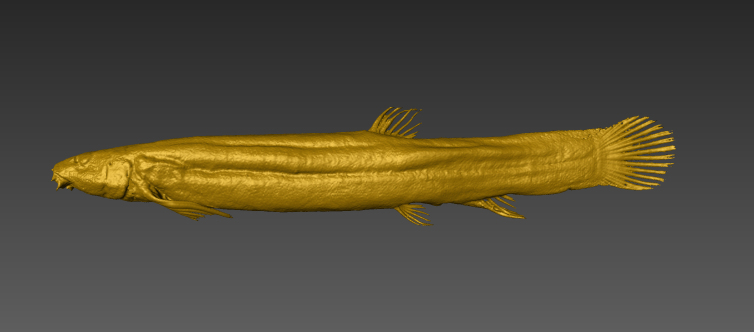
A 3D model of the surface of Misgurnus anguillicaudatus (Clade B1) (JNC342).

**Figure 5.**
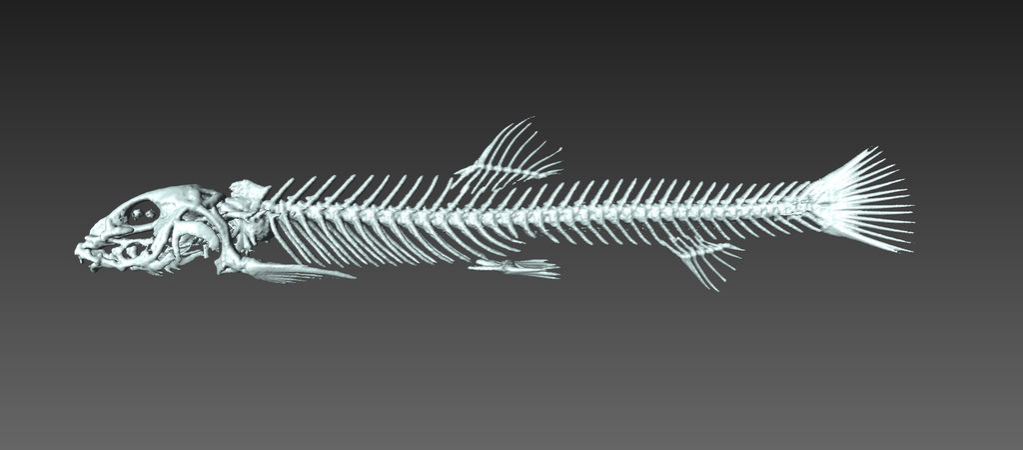
A 3D skeletal model of a paratype of Cobitis striata fuchigamii (MPM-Fi1501).

**Figure 6.**
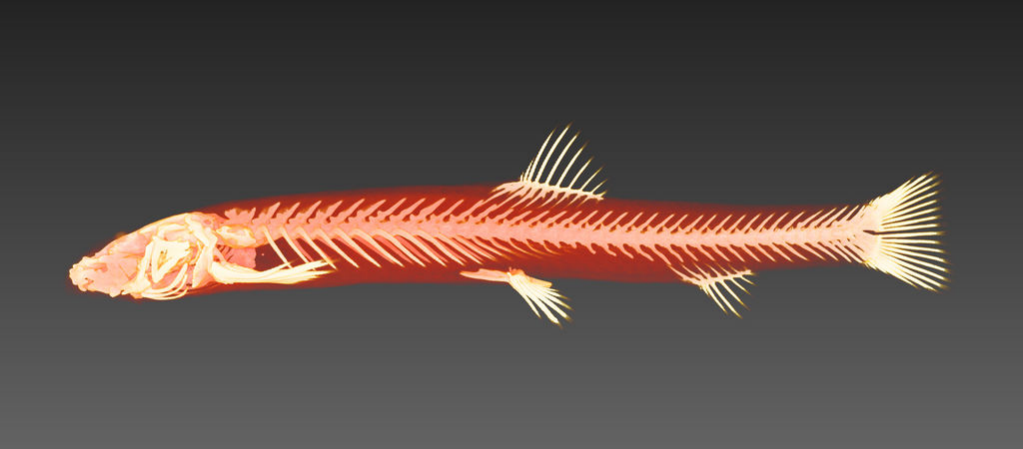
CT scanned data of Barbatula oreas (JNC256).

Photo images were taken in the field by capturing individuals (Fig. 1) (Kano and Nakajima 2014) and/or by snorkelling (Fig. 2). The formalin-fixed specimens were also photographed in the laboratory (Fig. 3) followed by CT scanning (Aloka Latheta LCT-200, Hitachi, Ltd.). 3D surface (Fig. 4; CT value: -400 to -40) and skeletal (Fig. 5; CT value: 5 to 200) models were extracted from the CT data. The CT data (Figs 6, 7) in raw file format were also stored and available on the web.

## Geographic coverage

### Description

Inland water habitats of Japan.

### Coordinates

23 and 46 Latitude; 150 and 123 Longitude.

## Taxonomic coverage

### Description

This paper includes all the known 35 loach taxa/clades (103 specimens) in Japan, of which 12 clades have still been undescribed or are uncertain.

**Type specimens**: The dataset includes nine type specimens as below.

Holotype: MPM-Fi1507 (Cobitis minamorii tokaiensis).

Paratypes: MPM-Fi1501 (Cobitis striata fuchigamii); MPM-Fi1502 (Cobitis striata hakataensis); MPM-Fi1503 (Cobitis kaibarai); MPM-Fi1504 (Cobitis magnostriata); MPM-Fi1505 (Cobitis minamorii minamorii); MPM-Fi1506 (Cobitis minamorii oumiensis); MPM-Fi1508 (Cobitis minamorii tokaiensis); MPM-Fi1509 (Cobitis minamorii saninensis).

**Undescribed/uncertain clades**: Below, we interpret the 12 undescribed/uncertain clades.

Twenty taxa of genus Cobitis have been hitherto known in Japan (Ikeda 1936, Okada and Ikeda 1939, Mizuno 1970, Kitagawa et al. 2003a, Kitagawa et al. 2003b, Suzawa 2006, Nakajima 2012, Nakajima et al. 2012, Nakajima 2016, Nakajima and Suzawa 2016, Nakajima and Uchiyama 2017), although five taxa are still undescribed/undecided without scientific names: One from Cobitis matsubarae species complex and the other four from Cobitis biwae species complex. Cobitis sp. "yamato" complex Type A (sensu Kitagawa et al. 2003a; one of Cobitis matsubarae species complex), distributed exclusively in Nagato District (western Honshu, Yamaguchi Prefecture), has peculiar genetic traits (Kitagawa et al. 2003a) and which should be treated as a single clade (Nakajima and Uchiyama 2017). It has a similar morphology to Cobitis matsubarae, whilst Nakajima and Uchiyama (2017) indicated that the genetic traits were close to Cobitis magnostriata and Cobitis takenoi. Amongst the four types of Cobitis biwae species complex, it is unknown which of these corresponds to the scientific name "Cobitis biwae" (Kitagawa et al. 2003b, Nakajima and Uchiyama 2017). Cobitis sp. BIWAE type A, also referred as "Western group (tetraploid form)" (Kitagawa et al. 2003b), is distributed amongst western Honshu, northern Shikoku, Awaji Island and eastern Kyushu. Cobitis sp. BIWAE type B, also referred to as "Western group (diploid form)" (Kitagawa et al. 2003b), is distributed in western Honshu and Oki-dougo Island. Cobitis sp. BIWAE type C, also referred to as "Eastern group" (Kitagawa et al. 2003b), is distributed between eastern Honshu and Sado Island. Cobitis sp. BIWAE type D, also referred to as "Kochi group" (Kitagawa et al. 2003b), inhabits the rivers flowing to Tosa Bay, southern Shikoku.

The situation about "Misgurnus anguillicaudatus" is rather complicated. Six taxa/clades of Misgurnus anguillicaudatus species complex has been hitherto known in Japan, namely "A", "B1", "B2", "Jindai", "IR" and "OK". Misgurnus sp. (Clade A) is a native loach clade of Japan, although the distribution is limited to northern parts such as Hokkaido and eastern Honshu (Morishima et al. 2008, Nakajima and Uchiyama 2017). Misgurnus anguillicaudatus (Clade B1) is another clade native to Japan and is widely distributed amongst Hokkaido (likely domestically introduced from Honshu), Honshu, Shikoku, Kyushu and several isolated islands (Morishima et al. 2008, Kano et al. 2011). Misgurnus anguillicaudatus (Clade B2) is a non-native clade introduced from mainland China and is also widely distributed amongst Honshu, Kyushu and Sado Island (Morishima et al. 2008, Kano et al. 2011). Misgurnus anguillicaudatus (Jindai), so-called "Jindai-dojyô" in Japanese, meaning "God's vicarious loach" or "loach in God's era", is an unusual local population that is already extinct. The loach had been distributed exclusively in Shindo Zone (Iga City, Mie Prefecture) having been visibly discriminated from the sympatric M. anguillicaudatus (Clade B1) by its size: the body size of the loach sometimes reached more than 30 cm (Takeda 1978). The ecology of the loach also seemed to be different from M. anguillicaudatus (Clade B1) as the trials of artificial breeding of the loach were extremely difficult and in vain, while the breeding of M. anguillicaudatus (Clade B1) was much easier (Takeda 1978). No genetic information on the loach was unfortunately available at present as the few old specimens were all formalin-fixed. Misgurnus sp. IR is distributed amongst several islands of Amami Islands and Iriomote Island, the southern part of Japan, with peculiar genetic/morphological traits (Shimizu et al. 2011, Kano et al. 2012, Nakajima and Uchiyama 2017). Misgurnus sp. OK has been found from Okinawa and Yonaguni Islands in Japan (Shimizu et al. 2011, Nakajima and Uchiyama 2017). The native distribution ranges of Misgurnus sp. IR and Misgurnus sp. OK are unknown.

Two Lefua species have still been left undescribed. Lefua sp. 1 is distributed amongst a narrow range of western Honshu, eastern Shikoku, Awaji Island and Shodo Island (Nakajima and Uchiyama 2017). Lefua sp. 2 is distributed in a limited area of Tokai region of Honshu, which is genetically discriminated from allopatric Lefua sp. 1 although the morphology of the two is rather similar (Nakajima and Uchiyama 2017).

**Non-native to Japan**:

Misgurnus anguillicaudatus (Clade B2): Definite native range of M. anguillicaudatus (Clade B2) is still unclear, but it potentially inhabits China (Chen 1981, Morishima et al. 2008), Korean Peninsula (Choi et al. 1990), northern Vietnam (Yen 1985) and Taiwan (Lin 2017).

Misgurnus dabryanus: Native to China (Chen 1981), Korean Peninsula (Choi et al. 1990) and Taiwan (Lin 2017).

Lefua costata: Native to northern China (Shedko et al. 2008, Miyazaki et al. 2011) and Korean Peninsula (Choi et al. 1990, Mihara et al. 2005, Shedko et al. 2008).

**IUCN rank and extinction**: Japanese loaches are generally faced with extinction and a part of them are listed in the IUCN Red List. Two loaches have been unconfirmed for the last 20 years indicating extinction.

CR: Parabotia curtus

NT: Cobitis takatsuensis

LC: "Misgurnus anguillicaudatus" that potentially includes Misgurnus sp. (Clade A), M. anguillicaudatus (Clade B1), M. anguillicaudatus (Clade B2), M. anguillicaudatus (Jindai), Misgurnus sp. IR and Misgurnus sp. OK.

Assumed to be extinct: Cobitis minamorii yodoensis; *Misgurnus anguillicaudatus *(Jindai).

### Taxa included

**Table:** 

Rank	Scientific Name	Common Name
kingdom	Animalia	Animals
phylum	Chordata	Chordates
subphylum	Craniata	Vertebrates and hagfishes
class	Osteichthyes	Bony fishes and tetrapods
subclass	Actinopterygii	Ray-finned fishes
order	Cypriniformes	Carps, loaches, minnows and relatives
family	Botiidae	Botiid loach
species	Parabotia curtus	"Ayumodoki"
family	Cobitidae	True loaches
species	Cobitis kaibarai	"Ariake-suji-shima-dojyô"
species	Cobitis magnostriata	"Oogata-suji-shima-dojyô"
species	Cobitis matsubarae	"Yamato-shima-dojyô"
species	Cobitis sp. "yamato" complex Type A	"Yamato-shima-dojyô" (Type A)
subspecies	Cobitis minamorii minamorii	"San'yô-kogata-suji-shima-dojyô"
subspecies	Cobitis minamorii oumiensis	"Biwa-kogata-suji-shima-dojyô"
subspecies	Cobitis minamorii saninensis	"San'in-kogata-suji-shima-dojyô"
subspecies	Cobitis minamorii tokaiensis	"Tôkai-kogata-suji-shima-dojyô"
subspecies	Cobitis minamorii yodoensis	"Yodo-kogata-suji-shima-dojyô"
species	Cobitis sakahoko	"Oyodo-shima-dojyô"
species	Cobitis shikokuensis	"Hina-ishi-dojyô"
species	Cobitis sp. BIWAE type A	"Oo-shima-dojyô"
species	Cobitis sp. BIWAE type B	"Nishi-shima-dojyô"
species	Cobitis sp. BIWAE type C	"Higashi-shima-dojyô"
species	Cobitis sp. BIWAE type D	"Tosa-shima-dojyô"
subspecies	Cobitis striata fuchigamii	"Onga-suji-shima-dojyô"
subspecies	Cobitis striata hakataensis	"Hakata-suji-shima-dojyô"
subspecies	Cobitis striata striata	"Chûgata-suji-shima-dojyô"
species	Cobitis takatsuensis	"Ishi-dojyô"
species	Cobitis takenoi	"Tango-suji-shima-dojyô"
species	Misgurnus sp. (Clade A)	"Kita-dojyô"
species	Misgurnus anguillicaudatus (Clade B1)	"Dojyô" (Japan clade)
species	Misgurnus anguillicaudatus (Clade B2)	"Dojyô" (China clade)
species	Misgurnus anguillicaudatus (Jindai)	"Jindai-dojyô"
species	Misgurnus sp. IR	"Shinobi-dojyô"
species	Misgurnus sp. OK	"Hyoumon-dojyô"
species	Misgurnus dabryanus	"Kara-dojyô"
species	Niwaella delicata	"Ajime-dojyô"
family	Nemacheilidae	Stone loaches
species	Barbatula oreas	"Fuku-dojyô"
species	Lefua costata	"Hime-dojyô"
species	Lefua echigonia	"Hotoke-dojyô"
species	Lefua nikkonis	"Ezo-hotoke-dojyô"
species	Lefua sp. 1	"Nagare-hotoke-dojyô"
species	Lefua sp. 2	"Tôkai-nagare-hotoke-dojyô"

## Temporal coverage

**Data range:** 1954-6-06 – 2017-12-15.

### Notes

Several specimens have no temporal information.

## Collection data

### Collection name

JNC/JNCP (J. Nakajima's personal collection); KPM (Kanagawa Prefectural Museum of Natural History); LBM (Lake Biwa Museum); MPM/MPMQ (Mie Prefectural Museum); OMNH (Osaka Museum of Natural History); QUYK (Y. Kano's personal collection); TKPM (Tokushima Prefectural Museum).

### Specimen preservation method

Fixed in formalin and preserved in 70% ethanol.

## Usage rights

### Use license

Other

### IP rights notes

Creative Commons Attribution Non Commercial (CC-BY-NC) 4.0 License

## Data resources

### Data package title

Photo images, 3D models and CT scanned data of loaches (Botiidae, Cobitidae and Nemacheilidae) of Japan

### Number of data sets

3

### Data set 1.

#### Data set name

loachesOfJapan

#### Data format

html; jpg

#### Number of columns

6

#### Character set

UTF-8

#### Download URL

http://ffish.asia/loachesOfJapan

#### Description

All the 35 known clades of loaches inhabiting Japan are listed with photo images. Below, the main 6 columns are listed;

**Data set 1.** 

Column label	Column description
Scientific name	Formal scientific name or tentative name
Taxon	Taxonomical hierarchy (order/family/genus)
Species image	Photo images of the species
N	Number of specimen/occurence data
Specimens/data distribution	Showing rough localities of the occurence on a map
Other information	Other information such as Japanese name

### Data set 2.

#### Data set name

loachesOfJapan3D

#### Data format

html; Wavefront object format (.obj); CT dicom file (.dcm)

#### Number of columns

12

#### Character set

UTF-8

#### Download URL

http://ffish.asia/loachesOfJapan3D

#### Description

Surface/skeletal 3D models and CT scanned data are available for all the clades (103 individuals). To render the CT dicom files as a visual 3D volume, several free software are available. Below, the main 12 columns are listed;

**Data set 2.** 

Column label	Column description
Specimen/Data ID	ID for the specimen/occurence
Images	Downloadable images/files of photos, 3D models and CT scanned data
Species	Scientific name (or tentative name) of the specimen
Taxon	Taxonomical hierarchy (order/family/genus)
N	Number of the individual(s)
DNA information	DNA sequence data if available
Location	Description of the locality
Specimens/data distribution	Showing rough localities of the occurence on a map
Sample year/month/day	Temporal infomation of the sampling
Japanese name	Japanese name
English name	English name or roman phonetics for Japanese
Comment	Other infomation such as sex, holotype, paratype etc.

### Data set 3.

#### Data set name

Photo images, 3D models and CT scanned data of loaches (Botiidae, Cobitidae and Nemacheilidae) of Japan

#### Data format

Darwin Core Archive

#### Number of columns

10

#### Download URL

http://ipt.pensoft.net/resource?r=jp_loaches_3dct_models

#### Description

GBIF registered occurrence data for the specimens. Below, the main 10 columns are listed;

**Data set 3.** 

Column label	Column description
occurrenceID	Occurrence ID and URL
basisOfRecord	The specific nature of the data record
eventDate	The date-time or interval during which the specimen collected
scientificName	Scientific name (or tentative name) of the specimen
decimalLatitude	Rough value of decimal latitude
decimalLongitude	Rough value of decimal longtitude
verbatimLocality	Description of the locality
typeStatus	Noted if the specimen is holotype or paratype
sex	Discrimination of male or female, while some are unknown
vernacularName	Japanese name

## Figures and Tables

**Figure 7.** A movie for CT scanned data of Cobitis minamorii yodoensis (OMNH-P45848), changing the camera angle and CT value.
